# Efficiency of the Coriolis µ Air Sampling Device for Fungal Contamination Analysis of Indoor Air: A Case Study

**DOI:** 10.3390/pathogens14040345

**Published:** 2025-04-03

**Authors:** Mohamad Al Hallak, Thomas Verdier, Alexandra Bertron, Myriam Mercade, Pascale Lepercq, Christine Roques, Jean-Denis Bailly

**Affiliations:** 1 College of Engineering and Technology, American University of the Middle East, Egaila 54200, Kuwait; mohamad.al-hallak@aum.edu.kw; 2 Laboratory of Materials and Durability of Construction, University of Toulouse, UPS, INSA, 31077 Toulouse, France; tverdier@insa-toulouse.fr (T.V.); bertron@insa-toulouse.fr (A.B.); 3 Toulouse Biotechnology Institute, University of Toulouse, CNRS, INRAE, INSA, 31077 Toulouse, France; mercade@insa-toulouse.fr (M.M.); lepercq@insa-toulouse.fr (P.L.); 4 Chemical Engineering Laboratory, University of Toulouse, CNRS, INPT, 31062 Toulouse, France; roques730@aol.com; 5 Laboratory of Agro-Industrial Chemistry, University of Toulouse, INRAE, INPT, 31030 Toulouse, France

**Keywords:** sampling method, impingement, indoor air, airborne fungal spores, Coriolis µ air sampler

## Abstract

Molds are frequent indoor contaminants, where they can colonize many materials. The subsequent aerosolization of fungal spores from moldy surfaces can strongly impact indoor air quality and the health of occupants. The investigation of fungal contamination of habitations is a key point in evaluating sanitary risks and understanding the relationship that may exist between the fungal presence on surfaces and air contamination. However, to date there is no “gold standard” of sampling indoor air for such investigations. Among various air sampling methods, impingement can be used for capturing fungal spores, as it enables real-time sampling and preserves analytical follow-up. Its efficiency varies depending on several factors, such as spore hydrophobicity, sampling conditions, etc. Sampling devices may also impact the results, with recovery rates sometimes lower than filtration-based methods. The Coriolis µ air sampler, an impingement-based device, utilizes centrifugal force to concentrate airborne particles into a liquid medium, offering flexibility for molecular analysis. Several studies have used this device for air sampling, demonstrating its application in detecting pollen, fungal spores, bacteria, and viruses, but it is most often used in laboratory conditions. The present case study, conducted in a moldy house, aims to investigate the efficiency of this device in sampling fungal spores for DNA analysis in indoor environments. The results obtained suggest that the use of this device requires an optimized methodology to enhance its efficiency and reliability in bioaerosol research.

## 1. Introduction

Molds represent a widespread group of fungal contaminants frequently encountered in indoor environments [1,2,3]. Within favorable conditions (water activity, nutrients availability, oxygen, etc.), these microorganisms can colonize a wide variety of materials, including wood, plasterboard, or textiles [1,4]. At a certain level of their growth, mold colonies release fungal spores and other fungal particles into the indoor air in a process known as aerosolization [5,6,7,8]. The presence of airborne fungal spores in indoor spaces poses notable health risks to occupants, ranging from mild allergic reactions to severe respiratory diseases such as asthma, hypersensitivity pneumonitis, fungal sinusitis, etc. [1,9,10]. Moreover, certain fungal species, belonging to *Aspergillus*, *Stachybotrys,* or *Penicillium* genera, produce mycotoxins, which may intensify their impact on human health [11,12,13]. The sanitary risks associated with fungal contamination highlight the urgent need for effective monitoring and analysis of indoor air quality.

Understanding the relationship between fungal growth on surfaces and the presence of airborne spores is critical for assessing exposure risks and developing intervention strategies (targeted cleaning and remediation, moisture control, fungal growth inhibition, etc.) [14,15]. However, this requires precise and reliable air sampling methods to quantify fungal spores and characterize their diversity. Despite significant advancements in air sampling techniques, a universal “gold standard” method for sampling indoor air is crucial [16,17].

Indeed, air sampling methods play a pivotal role in fungal contamination analysis, enabling the collection and analysis of bioaerosols effectively. These methods include filtration, impaction, sedimentation, and impingement techniques, each with its own advantages and limitations.

Among the various available approaches, impingement-based methods are widely used due to their ability to capture airborne particles in a liquid medium, preserving their viability for further analysis [18,19,20,21,22,23,24]. Such samples provide important information about the viability of the fungi in remediation or their infectious potential. This collection method not only preserves spore viability, but it also allows the realization of dilutions before cultivation or, on the contrary, the concentration needed to adapt the quantity of biological material for DNA extraction and molecular analysis [25,26]. The Coriolis µ air sampler represents a cutting-edge application of the impingement principle, utilizing centrifugal force to concentrate airborne particles into a liquid [27,28,29]. This device offers several advantages, including real-time sampling, adaptability to various analytical methods, and high sensitivity in detecting fungal spores, bacteria, viruses, and pollens [28].

Previous studies have demonstrated the versatility of the Coriolis µ air sampler in different contexts, including laboratory tests [28,30,31], wastewater treatment plants [32], and outdoor air quality monitoring [29]. However, its application in indoor environments, particularly in assessing fungal contamination in mold-affected buildings, remains underexplored. This study aims to address this gap by evaluating the efficiency of the Coriolis µ air sampler in capturing airborne fungal spores for DNA-based analyses in a mold-contaminated house. A comparison between air and surface fungal populations was conducted to analyze the relationship between the presence of moldy surfaces and indoor air quality.

## 2. Materials and Methods

### 2.1. Description of the House and Moldy Surfaces

**Figure 1:**
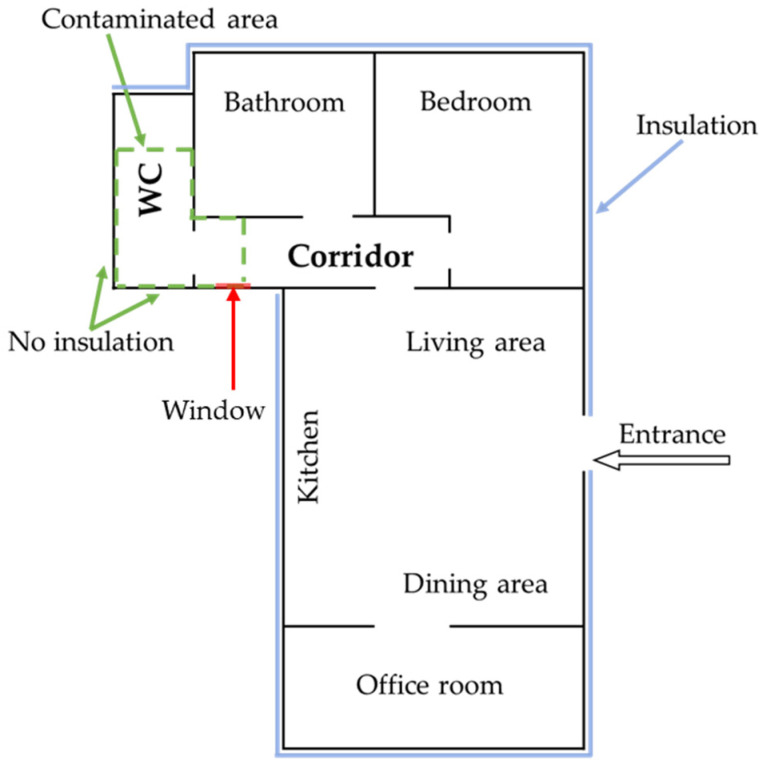
House plan indicating the location of the moldy area in the WC and the corridor (in green) and in the window (in red).

Sampling was carried out in an 80 m^2^ house in the village of Mont-Rosier (81) in the Occitanie region, France. The house, presented in Figure 1, is composed of one living room, one kitchen and one dining room, one office room, one bedroom, one bathroom, and one water closet (WC). The corridor, permitting access into the bathroom and the WC, as well as the WC presented visible fungal development.

### 2.2. Air Sampling

**Figure 2:**
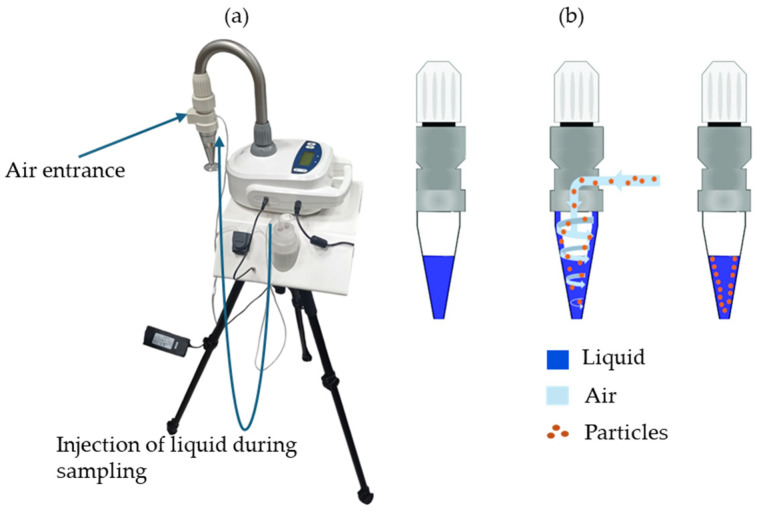
(**a**) Coriolis µ air sampler (Coriolis µ) with long-time monitoring (LTM) option; (**b**) impingement sampling principle.

Air sampling was carried out following the impingement principle using the Coriolis^®^ µ air sampler (Coriolis µ) with the long-time monitoring (LTM) option (Bertin Technologies; 78180 Montigny-Le-Bretonneux, France) (Figure 2) [27]. This technique uses cyclonic motions to collect airborne particles in a liquid solution (suspension) without passing through filters or sampling towards agar plates. The duration of air sampling using the Coriolis µ alone is limited to 10 min due to the losses of liquid from the sampling cone during air collection. With the LTM option, it can be extended up to six hours. This option allowed the conservation of liquid volume in the sampling cone through liquid injection into the cone during collection. In addition, the airflow rate of the device can be adjusted from 100 to 300 L/min. So, the maximum volume of air that can be collected using the Coriolis µ alone is 3 m^3^, while with the LTM option, up to 108 m^3^ of air can be collected.

In the collection cones, 15 mL of a phosphate buffer solution was supplemented with 0.05% of Tween 80 to help disperse the hydrophobic fungal spores in water, as the surfactant properties of Tween 80 can improve the wettability of hydrophobic surfaces [33].

After air sampling, samples were sent to the laboratory for analysis. Samples were collected in two zones, the WC (WA) and the corridor (CA).

The temperature (°C) and the relative humidity (%) were measured during sampling (performed in February 2022) using an HMT120 sensor supplied by VAISALA (Saclay, France) [34].

### 2.3. Surface Sampling

**Figure 3:**
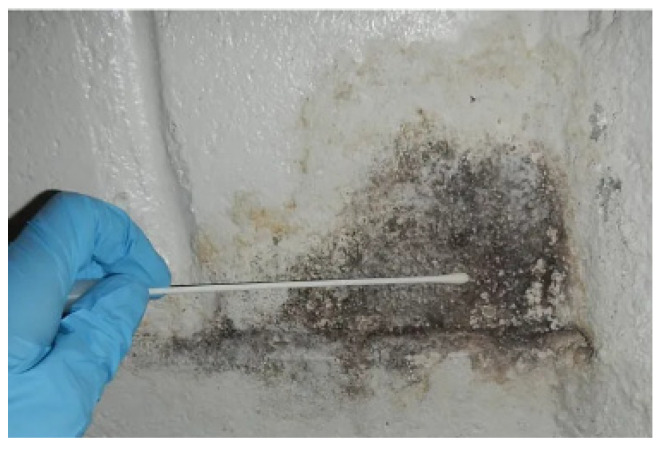
Moldy surface sampling using a sterile swab (template is not present on the photo).

In parallel to air sampling, moldy surface samples were also taken. For that, swab sampling was used. Sterile swabs (VWR; 93114 Rosny-sous-Bois Cedex, France) were humified with the neutralizing solution inside the tube, and a surface area of 5 × 5 cm^2^, visualized by a template, was sampled by gently scratching horizontally and vertically (Figure 3) [35]. Then, the swab was inserted into its tube and sent for analysis. The results of sampling on two surfaces are represented in the Results section: one from the wall in the WC (WS) and one from the corridor’s wall (CS).

### 2.4. DNA Analysis

DNA sequencing without preliminary culture was used to analyze the fungal contamination of the samples. It is based on the isolation and determination of DNA sequences that are specific to certain taxonomic groups. Two fungal Internal Transcribed Spacer regions (ITS1 and ITS2) [36,37] were used for the identification of fungal genera/species in the four collected samples (two surface samples and two airborne samples). The abundances presented in the Results section are relative to each individual sample and do not permit a quantitative comparison between the different samples.

#### 2.4.1. DNA Extraction

For air samples, the solution was gently deposited on 0.2 µm cellulose membrane filters (Sartorius). Then, all the liquid was filtered, and each filter was cut into four parts and placed two by two in a Tissuelyser 2 mL Eppendorf tube.

For surface samples, the buffer of the surface sample was transferred to the Tissuelyser 2 mL Eppendorf tube, and the terminal part of the swab was cut and placed in the same tube. Then, the tubes were centrifuged, and the supernatant was discarded. The pellet and swab of the fungal particles remained in the Tissuelyser 2 mL Eppendorf tube.

For all the tubes, DNA extraction was carried out using a ZymoBiomics DNA Miniprep Kit (Ozyme, Saint-Cyr-l’Ecole-France) according to the manufacturer’s instructions. After the Tissuelyser step, the recommended protocol was completed by 2 steps, 3 min in ice and 2 min at 60 °C. All steps were repeated twice, and the final elution was conducted in 100 µL.

DNA concentrations and purity were measured with a Nanodrop 2000 Strectrophotometer (Thermo Fischer Scientific, Breda, The Netherlands). DNA integrity was checked by 0.7% agarose gel electrophoresis, stained with an ethidium bromide bath, and visualized with a BioRad UV chemo doc imager (Marnes la Coquette, France).

#### 2.4.2. Amplification

The following DNA primers were used to amplify a region of the fungal ITS1: ITS1 F (5′-3′:TCCGTAGGTGAACCTGCGG) and ITS2 R (5′-3′: GCTGCGTTCTTCATCGATGC) and ITS2: ITS3 F (5′-3′: GCATCGATGAAGAACGCAGC and ITS4 R (5′-3′:TCCTCCGCTTATTGATATGC).

The PCR amplification reaction was performed with Phusion High Fidelity DNA polymerase (NEB, Ipswich, MA, USA) with 5 ng of extracted DNA and was carried out as follows: 1 min at 98 °C/30 cycles; 10 s at 98 °C; 20 s at 59 °C; 20 s at 72 °C; and final extension at 72 °C for 10 min.

After the bead purification, the DNA concentration was determined using Qubit 2.0 (Thermo Fischer Scientific, Breda, The Netherlands), and the quality was determined by migration with a Bioanalyzer 2100 (Agilent Technologies, Santa Clara, CA, USA).

Library preparation was performed with a Nextflex Cell-Free DNA seq kit and a Nextflex DNA barcodes for Ion platforms kit (Perkin Elmer, Paris, France) according to the manufacturer’s recommendations. The concentration of the libraries was determined using Qubit 2.0 (Invitrogen by life technologies, Carlsbad, CA, USA), and the quality of the PCR product was evaluated after migration on a Bioanalyzer 2100 (Agilent, Santa Clara, CA, USA). The libraries were pooled at the same concentration. According to the manufacturer recommendations, the Ion 510™, Ion 520™, and Ion 530™ Kits–Chef were used to prepare the template on a 520 Chip and sequence the pool of libraries using the Ion Chef™ and the Ion S5 Prime™ Sequencing Systems (Thermo Fischer Scientific, Breda, The Netherlands).

Raw sequences in fast format were preprocessed using the rANOMALY 1.0.0 package [38] based on the DADA2 [39], DECIPHER 2.28.0 [40], and phyloseq 1.44.0 [41] packages. ITS1 and ITS2 amplicons were processed separately where cutadapt [42] was used to keep sequences with specific primers (ITS1 or ITS2); then, primers were removed. Reads with N bases or a low phred quality score (less than or equal to 2) were eliminated and reads under 100 bp length were removed. The remaining sequences were dereplicated and denoised thanks to the DADA2 function. This process generated a raw abundance table. Amplicon sequence variants (ASVs) with an overall frequency below 0.005% were removed from the dataset.

Taxonomic affiliation of all ASVs was performed with the id-taxa function from the DECIPHER 2.28.0 package [40] using the UNITE v8.2 [43] and UTOPIA 2022 [44] databases. The latter lists the totality of ITS sequences deposited on NCBI. The joint use of the two databases allows the best possible taxonomic affiliation. In parallel, a phylogenic tree was generated. The abundance table, the taxonomy table, the phylogenetic tree, and the metadata table generated the final “phyloseq” object that we used for the rest of the analyses. Sequences that were not assigned to the fungi domain were removed. Visualizations were generated using rANOMALY functions. A stacked bar-plot was used to compare the community with the 20 most abundant taxa at different taxonomic levels. Alpha diversity indexes (Supplementary Figure S1), Specific Richness, and the Shannon index were calculated and plotted. Beta diversity visualizations (Supplementary Figure S2) were carried out with the Bray–Curtis index and MDS ordination. Sequencing and data analysis were performed using services provided by the GeT-Biopuces platform (TBI, Toulouse, France), a member of IBISBA-FR (www.ibisba.fr), the french node of the European research infrastructure IBISBA (www.ibisba.eu). 

## 3. Results

### 3.1. Description of Moldy Surfaces and Sampling Locations

**Figure 4:**
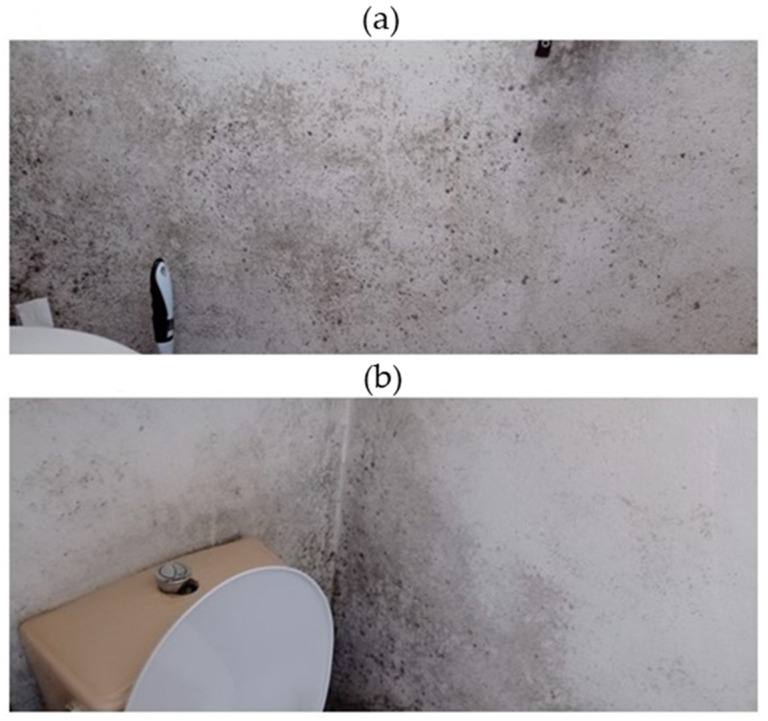
Appearance of the contaminated walls in the WC: (**a**) visual aspect of the upper part of the wall in the WC; (**b**) visual aspect of the wall and wall corner behind the WC bowl (February 2022).

**Figure 5:**
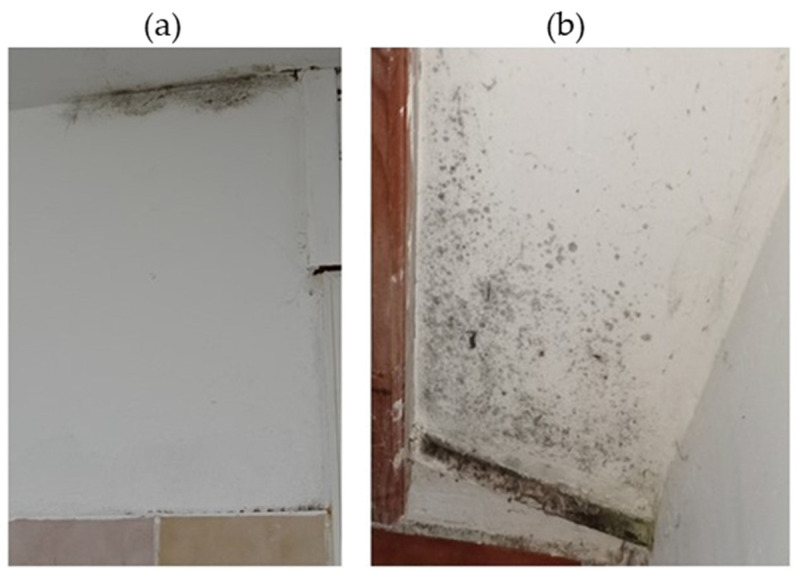
Visual aspect of the contaminated area in the corridor of the house: (**a**) upper edge of the wall in the corridor; (**b**) lower part of the wall in the corridor (February 2022).

Two rooms displayed visible mold development on the walls: the WC and part of the corridor. In these two rooms, the walls were made of painted concrete with no insulation materials. A window, used for ventilation, is located near the entrance of the WC. No visible fungal development was detected in other rooms of the house. In the WC (Figure 4), the visible contaminated area represented approximately 50% of the four walls (about 4.5 m^2^) (Table 1). In the corridor, the contaminated area was less than that in the WC and estimated to be about 0.7 m^2^ (Figure 5). Table 1 presents the dimensions, relative humidity, and temperature information of the rooms in the sampling locations.

### 3.2. Methodological Adaptation and Validation of Air Sampling

Table 2 presents the different conditions tested to define the adequate air sampling method. Initially, pretests on air sampling were conducted to investigate the necessary volume required to proceed to DNA extraction. The Coriolis µ air sampler was first used without the long-time monitoring (LTM) option, operating at its default maximum runtime of 10 min with an airflow rate of 300 L/min, resulting in a total sampled air volume of 3 m^3^. A 15 mL solution was used as the collection medium in the plastic cone.

During the sampling process, a significant loss of liquid from the plastic cone was observed, since by the end of the 10 min sampling period, the volume of liquid remaining in the cone was less than 1 mL.

To mitigate this issue, the airflow rate was progressively reduced. Despite this adjustment, considerable liquid loss persisted across all conditions (Table 2). At the lowest airflow rate (100 L/min), the remaining liquid volume after 10 min of sampling was approximately 5 mL, with 1 m^3^ of air collected per sample.

Then, three separate air samples were collected at 100 L/min for 10 min each, yielding 1 m^3^ of air per sample. However, DNA analysis could not be performed due to the insufficient quantity of collected DNA.

An increase in the volume of air sampled appeared necessary to collect enough particles for DNA analysis. To achieve this, the airflow rate was fixed at 100 L/min, as this setting resulted in the least liquid loss during sampling. Higher air volumes of 5, 10, and 15 m^3^ were collected by relaunching the sampling process every 10 min. Between each round, sterile liquid was manually added to the collection cone to compensate for losses and maintain sample integrity (Table 3).

For each targeted air volume, three independent samples were collected (n = 9) and subsequently analyzed. However, they did not provide a sufficient concentration of DNA to allow for its analysis (final DNA concentration < 5 ng/µL).

At this stage, the long-time monitoring (LTM) module was integrated into the sampling process. The use of LTM allowed liquid to be injected into the plastic cone at an adjustable flow rate to maintain a stable collection medium. It also prolonged the sampling duration, facilitating the collection of a larger quantity of airborne particles. To optimize the efficiency and reduce sampling time, the airflow rate was set at 200 L/min. Three different air volumes—24 m^3^, 30 m^3^, and 36 m^3^—were sampled to determine the minimum air volume required for DNA extraction. The liquid injection flow rate was adjusted to 1.2 mL/min to ensure consistent liquid refill throughout the sampling process. For each targeted air volume, three independent samples were collected (n = 9) and analyzed. DNA extraction was not feasible for the 24 m^3^ air samples, while the collected airborne particles appeared sufficient for DNA extraction for the 30 m^3^ and 36 m^3^ samples (Table 4).

Building upon the previous trials presented above; and to be sure of the collected DNA quantity, a sample volume of 36 m^3^ was collected for further airborne fungi analysis.

### 3.3. Identified Fungal Contaminants

#### 3.3.1. Airborne Fungal Contaminants

Air sampling in the rooms with moldy areas was therefore conducted using an airflow rate of 200 L/min and a running time of 3 h to collect 36 m^3^ of air. The final DNA concentrations obtained were 4.7 and 6.6 ng/µL for the WC and the corridor, respectively.

Table 5 presents the fungal genera identification from air samples based on the ITS1 and ITS2 regions separately. It must be noted that the high level of conservation of the ITS sequences within a fungal genus prevents the identification of the molds present at the species level. Indeed, the obtained sequences always matched at 100%, with several fungal species of one given genus.

Using ITS1, seventy-one different fungal genera were identified both in the WC and in the corridor. One genus only, out of the seventy-one detected, exceeded 10% of the total DNA: *Aspergillus* (12% in the WC, 15% in the corridor). The abundance of twenty genera ranged from 1–10%, among which most belonged to the *Basidiomycota* phylum (Table 5). The abundance of the other sixty-eight genera identified represented less than 1% of the total DNA, and they were grouped as “others”. Among the DNA present, 33.72% of that collected in the WC and 38.90% of that collected in the corridor did not match an identified genus.

Based on the ITS2 gene sequences, seventy-seven different fungal genera were identified both in the WC and in the corridor. The abundance of three genera, out of the seventy-seven detected, exceeded 10%: *Wallemia* (9.5% in the corridor, 21.5% in the WC), *Trametes* (12.6% in the corridor, 13.2% in the WC), and *Aspergillus* (8.4% in the WC, 13.3% in the corridor). The abundance of eight others ranged from 1 to 10% (Table 5). The abundance of the other sixty-six genera identified was less than 1%, and they were grouped as “others”. Among the collected DNA, 31.27% of that collected in the WC and 39.85% of that collected in the corridor was not identified using ITS2.

#### 3.3.2. Fungal Contaminants on the Surfaces

Table 6 presents the genera identification from the surface samples based on the ITS1 and ITS2 regions separately.

The number of fungal genera identified on the surface samples was much lower than that found in the air and mostly belonged to the *Ascomycota* phylum.

Indeed, using ITS1, only twelve different fungal genera were identified both in the WC and in the corridor. As for the air, *Aspergillus* was the dominant genus, representing 51% and 41% of the collected DNA in the WC and in the corridor, respectively. Another genus, *Cladosporium*, exceeded 10% of total DNA, with 30.8% of the DNA in the WC and 26% in the corridor. This fungal genus only represented a small percentage of the DNA collected form the air. A third genus represented a small percentage of the total DNA: *Debaryomyces* (1.8% in the WC, 3.3% in the corridor). The other nine genera identified all represented less than 1% of the DNA and are grouped as “others” in Table 6. Among the collected DNA, 20.78% of that from the WC and 23.88% of that from the corridor was not identified by ITS1 sequencing.

Based on the ITS2 gene sequences, twelve different fungal genera were also identified both in the WC and in the corridor. Contrary to the results obtained with ITS1, *Cladosporium* was the most prevalent genus, representing 60.5% of the DNA from the WC and 57% of that from the corridor. *Aspergillus* was second, with 30.7% of the DNA from the WC and 26% of that from the corridor. The abundance of two other genera ranged from 1 to 10%: *Debaryomyces* (6.9% in the WC, 9.8% in the corridor) and *Acremonium* (<1% in the WC, 1.9% in the corridor). The abundance of the other eight genera identified was less than 0.1%, and they are grouped as “others” in Table 6. Only 1.36% of the DNA collected in the WC and 4.84% of that collected in the corridor was not identified using ITS2.

## 4. Discussion

### 4.1. Methodological Adaptation of Air Sampling Protocol

The common methods used for air sampling include filtration, impaction, sedimentation, and impingement principles, each with its own advantages and disadvantages depending on the aim of the analysis. The filtration method is one of the most widely used techniques due to its high collection efficiency for airborne particles, including fungal spores and bacteria [17] and especially for long-duration sampling in low-concentration environments. Its supports culture-based and molecular analyses [16,45]. However, additional processing steps are required to extract biological material, which may lead to some biological material losses and viability reduction due to desiccation stress [18]. Additionally, in highly polluted environments, clogging issues may further affect sampling efficiency [46]. The impaction method, widely used in occupational health assessments [16], has shown high efficiency in high-concentration environments. Airborne particles are directly collected onto a solid medium, enabling immediate culture-based analysis [45]. However, using impaction, small-size bioaerosols may not be efficiently captured, and mechanical stress during collection can reduce microbial viability [19,46]. Additionally, it is unsuitable for microorganisms that are non-culturable on classical culture media and limited for molecular studies [18].

In addition to the above-mentioned techniques, air sampling following the impingement sampling technique enables the collection of airborne microorganisms into a liquid medium, preserving their viability for culture-based and molecular analyses. This method is particularly effective for real-time bioaerosol monitoring, making it highly suitable for studies requiring high sensitivity and advanced detection methods, such as qPCR-based microbial identification [18]. However, liquid losses at high airflow rates remain a significant challenge, potentially leading to variability in sample concentration and loss of collected microorganisms [19,20,23,26,47]. Additionally, the efficiency of impingement sampling relies on factors such as liquid composition, sampling conditions, and the types of microorganism targeted, necessitating careful optimization to ensure accurate results [46]. Moreover, it also requires precision in liquid injection rates for achieving consistent sample recovery over extended sampling periods.

Within this context, the aim of this study was to investigate the efficiency of the Coriolis µ air sampler with and without the LTM option as an impingement-based air sampling device for airborne fungal contamination molecular analysis in situ, using a moldy home as a case study.

Several laboratory studies have investigated the efficiency of the Coriolis µ air sampler in collecting different bioaerosols and reported various efficiencies of the process, particularly with respect to the airflow rate, sample integrity, microbial recovery, and particle sizes. For instance, Carvalho et al. [29] studied the physical efficiency of the Coriolis µ air sampler to collect airborne particles in the size range of 1–10 μm using the sampler along with surface membrane filters. The total sampled volume was 15 m^3^, collected after five sampling rounds of 3 m^3^ at a flow rate of 300 L/min per round. The highest collection efficiency of the device was 92%, obtained for 10 μm particles.

Another study by Chang et al. [48] evaluated the efficiency of this device targeting fungal spores. The airflow rate was 300 L/min, and the total volume of air sampled was 3 m^3^. In such conditions, the device efficiency was 80%. Additionally, Herve et al. [30] reported the necessity of sampling up to 20 m^3^ of air to successfully detect airborne swine *Influenza* A virus using Coriolis, suggesting that the smaller the particles are the lower the efficiency could be, and, therefore, the volume collected should be greater. By contrast, Ferguson et al. [26] recommended the use of this device after feasible molecular analysis of bacteria with only a 6 m^3^ sampling volume at 300 L/min. Rufino et al. [28] investigated the efficiency of the Coriolis µ air sampler with HEPA filters in targeting different bioaerosols. A sampling duration of 37 min, targeting 9.3 m^3^ of air at a 250 L/min flow rate, did not allow sample recovery, which is in accordance with our findings. To overcome this, they established a cumulative sampling process by replacing the collecting cone every ten minutes while sampling. Each collected sample was analyzed separately, and the results were aggregated to provide a cumulative measure of airborne particles over the entire sampling period. Sample recovery was improved by 50% using this strategy as opposed to the conventional continuous sampling technique.

Other air sampling methods used have been reported to require lower volumes than those defined in the current study, such as filter-based methods (1–10 m^3^) [49] and cyclone samplers (5–20 m^3^) [21].

However, all these studies were conducted in laboratory conditions in highly contaminated environments. There are only a few in situ studies that have been conducted using Coriolis µ air sampler.

In a study conducted by Mbareche et al. [50], they collected 9 m^3^ for air analysis using a molecular approach. But the sampling sites were at dairy farms and wastewater treatment plants, which are environments with a high level of aerosolized particles. This is also the case in a study of Viegas et al. conducted in poultry farms, where low air sampling volumes could be used to detect fungal contaminants [51].

In our case study, conducted in a house, a significantly higher volume of air (36 m^3^) was necessary to be able to conduct a molecular analysis of the airborne fungal population, probably related to the lower level of contamination despite the fact that the house displayed visible fungal growth on some walls. The need for such a high volume of air sampling may represent a strong limitation since the required volume may strongly exceed that of the sampled rooms, leading to the aspiration of air from other rooms or even from the outdoors in the case that an insufficiently insulated window is present. It is possible that the use of cultural methods instead of molecular ones allowed to decrease the required air volume. But a cultural approach would have limited the number of fungal genera identified to those able to grow under classic culture conditions. So, it appears that these two approaches shall be chosen according to the aim of the study. To evaluate the possible impact of fungal airborne contamination on health, the cultural approach may be sufficient since most of the pathogenic genera/species belong to the *Ascomycota* phylum and are able to grow on classic culture media, such as Malt Extract Agar or Sabouraud Dextrose Agar [52]. But if the aim is to investigate the fungal diversity and the relationships that may exist between indoor and outdoor air quality, then a molecular approach remains preferable due to its ability to also reveal the presence of uncultivable fungi [53].

The large amount of air required to capture fungal bioaerosol may also be related to the size of the targeted particles. Indeed, the Coriolis was found to have increased efficiency with particles larger than fungal spores [29]. So, the collection of particles usually ranging from 2 to 6 µm, such as fungal spores, may require an increased volume of air, especially to obtain a sufficient amount of DNA for molecular analysis.

So, with respect to the use of the Coriolis along with the LTM option, although it was found to be significant, as it provided a longer sampling duration and the automatic injection of liquid into the plastic cone, the high volume requirement (36 m^3^) raises concerns about its usability in the field for the DNA analysis of air samples.

### 4.2. Use of ITS1 and ITS2 for Fungal Contamination Analysis

The ability of ITS1 and ITS2 regions to identify fungal genera from surface and airborne samples has been widely studied, with both regions showing strengths and limitations depending on the context [54,55]. The results obtained in our study from surface samples showed a global similarity of the number of fungal genera detected using ITS1 or ITS2 but with sometimes great differences in their relative proportion. As an example, using ITS1, *Aspergillus* represented more than 50% of the total DNA collected on the walls of the WC (most abundant genus), whereas with ITS2, it only represented 30% (moving to second place, behind *Cladosporium*). Moreover, ITS2 appeared able to identify a larger part of the collected DNA (98.64% in the WC, 95.16% in the corridor) than the ITS1 region (79.22% in the WC, 76.12% in the corridor). The results obtained from the airborne samples showed a similar trend between ITS1 and ITS2. Once again, the proportion of the identified genera could strongly vary. For instance, using ITS1, the relative abundance of *Wallemia* was 2.43% and 2.18% in the WC and the corridor, respectively, whereas using ITS2, this genus represented 21.46% and 9.42% of the DNA collected from the WC and the corridor, respectively. Once again, ITS2 led to the identification of more fungal genera than ITS1 (77 vs 71). But, contrary to what was observed with surface samples, the proportion of unidentified DNA was much more important for both genes, representing from 30 to 40% of the airborne collected DNA. This could be due to the great diversity of the airborne fungal population.

Previous studies have reported similar observations regarding the comparability of ITS1 and ITS2. For instance, Blaalid et al. [56] noted that while both regions can yield similar taxonomic results, their performance may differ in terms of sensitivity to specific fungal groups or environmental conditions. In the same way, ITS2 has been reported to perform better in detecting fungal communities on surfaces, potentially due to its ability to provide more precise taxonomic resolution for certain groups [57], which is in accordance with our findings.

This study emphasizes that, while ITS1 and ITS2 are globally comparable in identifying genera, their differential performance across sample types underscores the importance of selecting the appropriate marker region based on the objectives of the analysis and environmental context.

However, the high proportion of unidentified DNA raises a question on the efficiency of these two most frequently used regions for airborne fungal genera molecular identification.

Moreover, the very strong conservation of these sequences between species within a given fungal genus makes the identification to the species level difficult, which could be of importance while investigating the potential impact of airborne fungal particles on human health. Indeed, obtained sequences often match at 100% with several distinct species, making it impossible to determine which one is present [58,59].

So, it appears necessary to select the target genes as a function of the investigated genera/species and the importance of the level of identification. As an illustration, Calmodulin and/or β-tubulin could represent good targets for *Aspergillus* species identification [60,61].

### 4.3. Comparison Between Surface and Airborne Fungal Populations

The results obtained show the dominance of *Aspergillus* and *Cladosporium* genera on surfaces both in the WC and in the corridor. The identification of these two genera on visibly contaminated surfaces is in accordance with previous studies investigating fungal growth on contaminated materials indoors [36,62,63,64,65,66]. Indeed, these two genera, belonging to *Ascomycota,* were often reported to be predominant on surfaces of building materials [67,68]. The abundance of *Aspergillus* and *Cladosporium* genera on surfaces may be explained by the ability of some species that belong to these genera, such as *Aspergillus flavus*, *Aspergillus versicolor*, *Aspergillus niger,* and *Cladosporium sphaerospermum,* to grow at a low water activity range (0.75–0.85) [69,70,71,72].

These two genera are of importance since they are both known as potent allergenic fungi. The *Aspergillus* genus also groups some species know, as possible pathogens and/or mycotoxin producers (*A. flavus, A. niger, A. fumigatus, A. versicolor*, etc.). So, their presence on indoor surfaces may lead to a deterioration of indoor air quality in the case of aerosolization and health problems following inhalation.

It is therefore of interest to note that *Aspergillus* was also one of the most abundant genera found in the air samples. This clearly highlights the relation that may exist between fungal development on indoor surfaces and air quality. By contrast, *Cladosporium* was much less predominant in the air samples, which is relevant to the differential organization of these two fungi. Indeed, in *Aspergillus*, conidia are small and bore by conidial heads as long chains that are easy to aerosolize [73]. In *Cladosporium*, conidia are bigger and often grouped, which makes them heavier and more difficult to remove from contaminated walls. The release of *Cladosporium* conidia from a colony is more related to relative humidity rather than air movement [74,75].

The analysis of the air samples also showed the presence of many genera that were not found on surfaces. They mostly belonged to the *Basidiomycota* phylum. This result is consistent since most of these fungal genera are not able to colonize building materials. Their presence also highlights the importance of outdoor air quality and the possible exchanges that occur between outdoor and indoor air. Indeed, most of the identified genera correspond to ones that are common in outdoor environments and especially in the countryside. The demonstration of the abundance and diversity of *Basidiomycetes* in the air was made possible thanks to the molecular approach used. Indeed, most of these genera, contrary to *Aspergillus* and *Cladosporium*, would not have been detected using the cultural approach.

Even if basidiomycetes are rarely involved in human pathologies, some reports have described the role of some genera/species in allergic reactions or fungal infections. For instance, the genus *Wallemia*, which was the most abundant in the air of the WC and was the second most abundant in the corridor using ITS2, has been associated with farmer’s lung disease and some rare cases of cutaneous infection [76].

In the same way, *Naganishia albida* was recently found to be involved in some human cutaneous infections [77]. This fungal genus was found to be predominant among basidiomycetes both in the WC and the corridor using ITS1 sequencing.

So, even if the occupants of the studied home did not present any health problems, probably linked to the fact that they rarely occupy this accommodation, which is a second home, several identified genera can be of health significance, and this suggests that remediation procedures should be undertaken to remove them from the indoor environment.

## 5. Conclusions

The aim of this study was to investigate the efficacy of the Coriolis μ air sampler, an impingement-based air sampling device, as an air sampling method for the determination of fungal airborne contamination using DNA analysis. It revealed that, in real conditions, a high volume of air is required to achieve feasible DNA analysis, even in a macroscopically moldy dwelling, highlighting a limitation of this device in specific room air sampling when DNA analysis is desired. The fungal identification conducted using both ITS1 and ITS2 region sequencing showed that a quite high proportion of DNA could not be identified, especially in the air samples, where fungal diversity appeared very important. Even if they are the most commonly used regions for fungal molecular identification, it seems that it would be of interest to test the ability of other genes to identify more accurately fungal genera that may be present in the air. Finally, *Aspergillus* was the predominant genus on surfaces and one of the most important also in the air, demonstrating the link between surface and air contamination and the importance of monitoring fungal growth in buildings to ensure indoor air quality.

## Figures and Tables

**Table 1:** Dimensions, relative humidity, and temperature data in the sampling location and the names of the surface and airborne samples.

House Part	Floor Space (m^2^)	Length (m)	Width (m)	Height (m)	Estimated Moldy Surface (m^2^)	Volume (m^3^)
WC	1.31	1.75	0.75	2.2	4.5	3
Corridor	6.63	3.9	1.7	2.55	0.7	17

**Table 2:** Summary of different airborne samplings carried out in step 1 to adapt the air sampling methodology.

Airflow Rate (L/min)	Sampling Duration (min)	Air Volume Sampled (m^3^)	Initial Liquid Volume in Sample (mL)	Remaining Liquid Volume in Sample (mL)
300	10	3	15	<1
250		2.5		1–2
200		2		2
150		1.5		3–3.5
100		1		4–5

**Table 3:** Summary of different airborne samplings carried out in step 2 to adapt the air sampling methodology.

Airflow Rate (L/min)	Sampling Duration (min)	Air Volume Sampled (m^3^)	Initial Liquid Volume in Sample (mL)	Added Volume per Round (mL)	Number of Rounds
100	50	5	15	10	5
	100	10			10
	150	15			15

**Table 4:** Air sampling carried out with LTM.

Airflow Rate (L/min)	Sampling Duration (min)	Air Volume Sampled (m^3^)	Initial Liquid Volume in Sample (mL)	Injection of Liquid from LTM (mL/min)	Volume Injected from LTM (mL)	DNA Concentration (ng/µL)
200	120	24	15	1.2	144	<5
	180	30			180	≈5
	240	36			216	≥5

**Table 5:** Relative abundance of genera, expressed in % of total DNA, along with the corresponding phylum identified from air samples using ITS1 or ITS2 region sequencing.

ITS1			
Phylum	Genus	Relative Abundance in the WC (% of Collected DNA)	Relative Abundance in the Corridor (% of Collected DNA)
*Basidiomycota*	*Naganishia*	5.39	4.73
	*Strobilurus*	5.23	4.73
	*Sterigmatomyces*	5.13	4.13
	*Cerrena*	3.28	1.55
	*Phanerochaete*	2.43	2.62
	*Wallemia*	2.43	2.18
	*Steccherinum*	2.22	1.39
	*Phellinus*	0.51	2.15
	*Trametes*	1.82	1.76
	*Antrodiella*	0.43	1.74
	*Sistotremastrum*	0.15	1.59
	*Coprinellus*	nd	1.45
	*Phlebia*	nd	1.27
	*Stereaceaegenera*	1.20	0.27
	*Corticium*	1.09	nd
	*Scytinostromella*	1	nd
*Ascomycota*	*Aspergillus*	12.03	14.98
	*Stemphylium*	3.45	nd
	*Penicillium*	1.31	1.98
	*Paraphoma*	nd	1.22
	*Cladosporium*	0.86	0.39
*Mucoromycota*	*Mucor*	1.7	0.18
	Others *	11.10	7.96
Unidentified fungal phylum	Unidentified fungal genera	37.24	41.73
**ITS2**			
**Phylum**	**Genus**	**Relative Abundance in the WC** **(% of Collected DNA)**	**Relative Abundance in the Corridor** **(% of Collected DNA)**
*Basidiomycota*	*Wallemia*	21.46	9.42
	*Trametes*	13.19	12.57
	*Sterigmatomyces*	2.56	1.85
	*Steccherinum*	2.49	0.22
	*Malassezia*	0.3	2.26
	*Phanerochaete*	1.38	2.22
	*Strobilurus*	1.81	1.08
*Ascomycota*	*Aspergillus*	8.35	13.33
	*Cladosporium*	2.5	3.65
	*Penicillium*	1.03	1.71
	*Debaryomyces*	0.78	1.17
	Others *	12.88	10.67
Unidentified fungal phylum	Unidentified fungal genera	31.27	39.85

*: Fungal genera grouped as “others” are detailed in Appendix A. nd: not detected.

**Table 6:** Relative abundance of genera identified, expressed in % of total DNA, from surface samples using ITS1 or ITS2 region sequencing.

ITS1			
Phylum	Genus	Relative Abundance in the WC (% of Collected DNA)	Relative Abundance in the Corridor (% of Collected DNA)
*Ascomycota*	*Aspergillus*	51.03	41.14
	*Cladosporium*	26.03	30.8
	*Debaryomyces*	1.81	3.27
	Others *	0.35	0.91
Unidentified fungal phylum	Unidentified fungal genera	20.78	23.88
**ITS2**			
**Phylum**	**Genus**	**Relative Abundance in the WC ** **(% of Collected DNA)**	**Relative Abundance in the Corridor** **(% of Collected DNA)**
*Ascomycota*	*Cladosporium*	60.47	57.31
	*Aspergillus*	30.69	25.98
	*Debaryomyces*	6.90	9.84
	*Acremonium*	0.45	1.87
	Others *	0.13	0.16
Unidentified fungal phylum	Unidentified fungal genera	1.36	4.84

*: Fungal genera grouped as “others” are listed in Appendix B.

## Data Availability

The data presented in this study are available upon request to the corresponding author.

## Supplementary Materials

The following supporting information can be downloaded at: https://www.mdpi.com/article/10.3390/pathogens14040345/s1, Figure S1: Alpha diversity of air and surface samples; Figure S2: Beta diversity of analyzed samples.

## Appendix A. Fungal Genera Identified in Air Samples and Grouped as “Others” in Table 5

○ List of the 50 other genera identified in the air samples using the ITS1 region: Fairmania, Phialophora, Epicoccum, Blumeria, Aureobasidium, Lewia, Debaryomyces, Acremonium, Thermomyces, Stereocaulon, Talaromyces, Torula, Verrucaria, Neosetophoma, Helgardia, Alatospora, Hyphodermella, Stereum, Psathyrella, Fuscoporia, Xylodon, Vuilleminia, Lyomyces, Crepidotus, Sistotrema, Phlebiopsis, Ceriporiopsis, Mycoacia, Vishniacozyma, Ceriporia, Postia, Tubaria, Coniophora, Hymenochaete, Sidera, Lenzites, Filobasidium, Schizophyllum, Skeletocutis, Gloeocystidiellum, Gloeophyllum, Bjerkandera, Udeniomyces, Dioszegia, Antrodia, Malassezia, Rhodosporidiobolus, Clathrus, Tubulicrinis, and Cutaneotrichosporon.
○ List of the 66 other genera identified in the air samples using the ITS2 region: Fairmania, Blastobotrys, Verrucocladosporium, Arthrobotrys, Fusarium, Trichoderma, Cephalotrichum, Pectenia, Rhinocladiella, Paraphoma, Saccharomyces, Mollisia, Thelebolus, Thermomyces, Talaromyces, Devriesia, Verrucaria, Clavariopsis, Cercospora, Neodevriesia, Cadophora, Coprinellus, Cryptococcus, Naganishia, Stereum, Peniophora, Hyphodermella, Dioszegia, Symmetrospora, Xylodon, Gloeophyllum, Ganoderma, Scytinostromella, Cerrena, Sistotrema, Sistotremastrum, Baeospora, Fuscoporia, Corticium, Phlebia, Hymenochaete, Xenasmatella, Antrodiella, Phellinus, Mycoacia, Filobasidium, Cortinarius, Ceriporia, Porostereum, Brevicellicium, Vishniacozyma, Hyphodontia, Myxarium, Byssomerulius, Clathrus, Fomitopsis, Phaeophlebiopsis, Polyporus, Tranzscheliella, Ceriporiopsis, Mycena, Stropharia, Moniliella, Skeletocutis, Lenzites, and Mucor.

## Appendix B. Fungal Genera Identified on Surface Samples and Grouped as “Others” in Table 6

○ List of the nine other genera identified on surfaces using the ITS1 region: Penicillium, Acremonium, Naganishia, Sterigmatomyces, Phanerochaete, Steccherinum, Wallemia, Vishniacozyma, and Clathrus.
○ List of the eight other genera identified on surfaces using ITS2 region: Penicillium, Fusarium, Trametes, Wallemia, Sterigmatomyces, Malassezia, Corticium, and Clathrus.
