# Supplementary material for: Efficiency of the Coriolis µ Air Sampling Device for Fungal Contamination Analysis of Indoor Air: A Case Study
Source: Pathogens. 2025 Apr 3;14(4):345. doi: 10.3390/pathogens14040345 (PMC12030049; doi:10.3390/pathogens14040345)
Supplement: Supplementary file 1 [file pathogens-14-00345-s001.zip › pathogens-3555190-supplementary.pdf]

Supplementary Materials

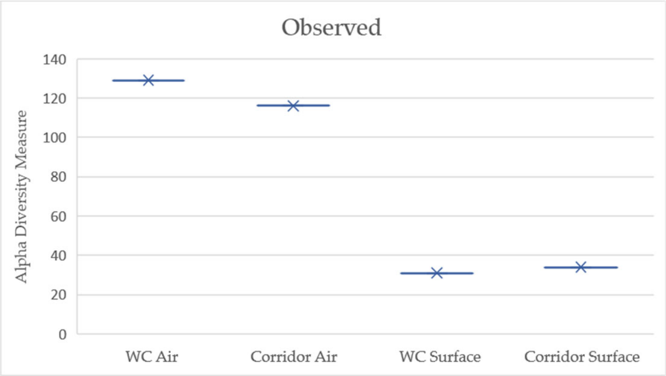

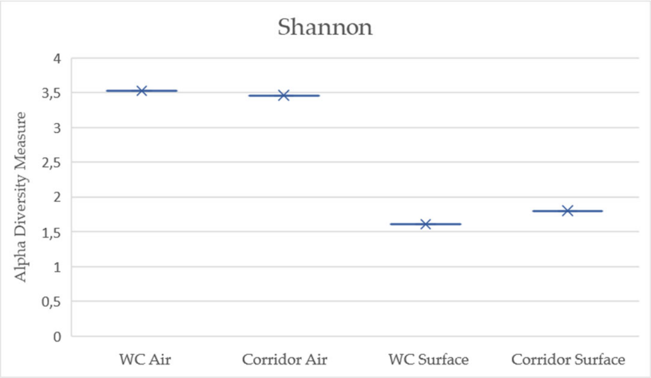

Supplementary Figure S1: Alpha diversity of air and surface samples.
Alpha diversity allows us to determine the specific richness of the microbial communities in each sample. Several indices exist, but here is presented the raw specific richness, i.e. the number of ASV present in the samples. The Shannon index takes into account both the richness and relative abundance of each ASV in order to assess the balance of the microbial community. For an equal number of ASVs, a balanced community will have a higher Shannon index than one with a very dominant taxon compared to the rest of the community.

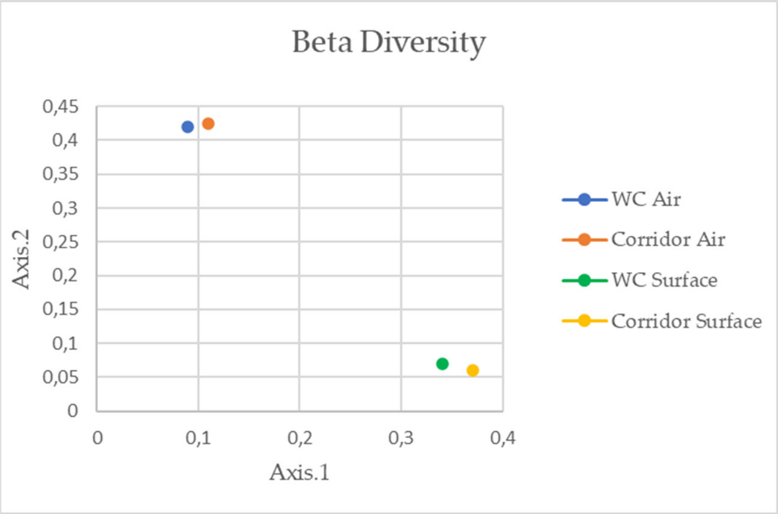

Supplementary Figure S2: Beta diversity of analyzed samples.
Beta diversity uses a distance matrix (BrayCurtis distance, for instance) calculated from the abundance table to determine differences in microbial community composition between samples. Ordination plots like the MDS display samples on an orthogonal space. The closer the samples are on the ordination, the more similar their communities are.

We can see that, for both alpha and beta diversity, the air or surface samples are close to each other, but, by contrast, the two groups are far apart, which is consistent with the obtained results regarding present fungal diversity. Air samples have a greater fungal diversity than surface samples.
